# Reorganization of spatial maps in the hippocampal circuit

**DOI:** 10.1186/1471-2202-12-S1-P340

**Published:** 2011-07-18

**Authors:** Federico Stella, Alessandro Treves

**Affiliations:** 1 Cognitive Neuroscience Sector, SISSA, Trieste, 34136, Italy; 2 Kavli Insitute for Systems Neuroscience and Centre for the Biology of Memory, NTNU, Trondheim, Norway

## 

It is known that the hippocampus plays a central role in the storage and in the retrieval of episodic memories. In particular the study of population dynamics in hippocampal place cells has emerged as one of the most powerful tools for understanding the encoding, storage and retrieval of episodic memories. Place cells are hippocampal neurons whose discharge is strongly related to a rat location in its environment. The existence of place cells has led to the proposal that they are part of an integrated neural system, which involves also parahippocampal regions, dedicated to spatial navigation and memory. Accumulating evidence suggests that environments are generally represented in hippocampal cells as a collection of manifolds associated to real space. Observed phenomena like global remapping and rate remapping can give us insights on the nature of these maps and on the attractor dynamics that governs their storage and retrieval.

As the representation of the same environment is differently expressed in hippocampal subregions it becomes important to understand the function of the sequential transmission through the DG, CA3 and CA1. Indeed, while the particular autoassociative operations are ascribed mainly to the recurrent CA3 network, the role of CA1 and of the CA3-to-CA1 connections is not clear.

We address these questions, restricted to Schaffer Collaterals connections for clarity, within a simplified mathematical network model. The model network simulates the storage on CA3 of one or more spatial representations, and their transfer to CA1. We quantify through information measures the ability of Schaffer Collaterals connections to reproduce the retrieved representation in CA1 and with which modifications, after a training phase in which they are modified through model Hebbian plasticity, or else after having been structured top-down. In particular we analyzed the way in which correlated or uncorrelated CA3 maps are actually represented in CA1. We find that in the CA1 maps there is a “smoother” representation of space, and that there is substantial difference in the way information is expressed. Finally, we find that even networks of considerable size can only approximate the idealized notion of a 2D quasi-continuous dynamical attractor.

